# PD-L1^+^ CD49f^+^ CD133^+^ Circulating tumor cells predict outcome of patients with vulvar or cervical cancer after radio- and chemoradiotherapy

**DOI:** 10.1186/s12967-025-06277-w

**Published:** 2025-03-13

**Authors:** Selina Gies, Patrick Melchior, Istvan Molnar, Gregor Olmes, Russalina Stroeder, Tanja Tänzer, Maike Pohlers, Moritz Schäfer, Laura Theobald, Martina Sester, Erich Franz Solomayer, Barbara Walch-Rückheim

**Affiliations:** 1 Center of Human and Molecular Biology (ZHMB) Kirrbergerstraße, Institute of Virology, Saarland University, Building 47, D-66421 Homburg/Saar, Germany; 2 Department of Radiation Oncology, Saarland University Medical Center, Homburg/Saar, Germany; 3 Department of Obstetrics and Gynecology, Saarland University Medical Center, Homburg/Saar, Germany; 4 Department of Transplant and Infection Immunology, Saarland University, Homburg/Saar, Germany; 5 Experimental Gynaecological Oncology, Gynecology, Faculty of Medicine, University of Augsburg, Augsburg, Germany

**Keywords:** Circulating tumor cells, Vulvar cancer, Cervical cancer, Radiotherapy, Chemoradiotherapy

## Abstract

**Background:**

Monitoring individual therapy responses of patients with cancer represents a major clinical challenge providing the basis to early identify metastases and cancer relapse. We previously demonstrated that radio- or chemoradiotherapy affects the systemic cellular milieu of patients with vulvar or cervical cancer and creates individual post-therapeutic environments associated with cancer relapse. Circulating tumor cells (CTCs) in the systemic milieu are related to metastases and relapse; however, their quantitative and phenotypic characteristics during therapy of patients with vulvar and cervical cancer are still unknown.

**Methods:**

In this prospective, longitudinal study, we verified the presence of CTCs via immunofluorescence and systemically characterized CTCs by flow cytometry from the blood of 40 patients with vulvar and 115 patients with cervical cancer receiving surgery, adjuvant radiotherapy (aRT), chemoradiotherapy (aCRT) or primary chemoradiotherapy (pCRT) and linked the presence of different CTC subpopulations with individual outcome of disease.

**Results:**

Pre-therapeutic cytokeratin^+^ CD45^−^ CTC numbers significantly correlated with tumor FIGO stages, lymph node metastases and relapse. While surgery only did not significantly alter CTC occurrence, aRT and aCRT as well as pCRT differentially decreased or increased CTCs in patients with both tumor entities compared to baseline levels. Therapy-mediated increased CTC numbers were directly linked with subsequent cancer recurrence on follow-up. Phenotypic characterization of CTCs revealed enhanced expression of the stem cell marker CD133 as well as the integrin α6 (CD49f) after aRT, aCRT and pCRT. Furthermore, the aRT, aCRT and pCRT cohorts exhibited increased proportions of Programmed Cell Death Protein Ligand (PD-L1) expressing cells among post-therapeutic CTCs. Notably, post-therapeutic PD-L1^+^ CD49f^+^ CD133^+^ numbers ≥ 5/ml in patients with vulvar cancer and ≥ 2/ml in patients with cervical cancer were associated with reduced recurrence-free survival on follow-up.

**Conclusion:**

Our study unravels individual therapy-induced changes in CTC phenotypic characteristics and occurrence in the patients’ blood and their association with cancer relapse. Our results may help to explain differences in the individual courses of disease of patients with vulvar and cervical cancer and suggest PD-L1, CD49f and CD133 as targets for immunotherapy in vulvar and cervical cancer.

**Supplementary Information:**

The online version contains supplementary material available at 10.1186/s12967-025-06277-w.

## Background

Treatment of metastatic vulvar or cervical cancers remains a major clinical challenge. Regarding gynecological malignancies, cervical cancer is the fourth most common cancer in woman worldwide with 661,021 new cases and 348,189 cancer-related deaths in 2022 [1]. Meanwhile, vulvar cancer is a relatively rare tumor with 47,342 new cases and 18,579 cancer-related deaths in 2022 worldwide [1] representing only 2–5% of all gynecological malignancies [2]. Both tumor entities can be linked to persistent infection with high-risk human papillomavirus (HPV).

More than 90% of cervical cancers develop as a consequence of persistent infection with high-risk human papillomavirus (HPV) [3]. Guideline-based therapies for cervical cancers depend on the disease stages according to the International Federation of Gynecology and Obstetrics (FIGO) classification. Besides surgery alone in early stages, therapeutic approaches include adjuvant platinum-based concurrent chemoradiotherapy (CRT) in addition to surgery for advanced cervical cancers with prognostic risk factors for local relapse, and primary CRT for locally advanced often inoperable cervical carcinoma (FIGO > IIB) [4].

In contrast to cervical cancers, approximately 43% of vulvar cancers worldwide are related to HPV infections [5]. Non-keratinized vulvar squamous cell carcinomas are often associated with HPV and mainly affect younger women [6] while keratinized vulvar squamous cell carcinomas affecting older women are usually HPV-independent and result from a chronic genital inflammatory disease, such as lichen sclerosus [7]. For vulvar cancers, most patients are primarily treated with a wide local excision of the primary tumor, often accompanied by adjuvant radiotherapy. Early-stage vulvar cancers were treated with radical excision of the primary tumor and adjuvant radiotherapy (aRT) to groins and pelvis is applied in case of advanced nodal involvement [8].

However, the initial tumor stage as well as positive lymph nodes affect the course of disease of patients with cervical or vulvar cancer. Depending on the initial tumor stage, 8–26% of women with cervical cancer experience relapse, most commonly within the first two years of completing primary treatment [9]. This results in a 5-year survival ranging from over 90% if diagnosed in an early, localized stage to less than 20% if diagnosed as distant or metastatic [10]. For patients with locally advanced vulvar tumors and positive lymph nodes prognosis remains poor, as 46% of these patients developed cancer recurrence after ten years [11]. In patients with operable vulvar cancer without lymph node involvement, the 5-year overall survival (OS) rate is 90%. However, in patients with nodal involvement, the 5-year OS rate is approximately 25–41% [12]. Thus, therapeutic management and early identification of metastatic cervical or vulvar cancer remains a major clinical challenge.

During metastases, some cancer cells detach from the primary tumor to reach the blood or lymphatic system and become circulating tumor cells (CTCs). In patients with cervical cancer, CTCs were defined as CD45^−^ cytokeratin^+^ cells [13]. To leave primary tumors CTCs pass through epithelial-mesenchymal-transition (EMT) receiving the capability to target distinct organs and contribute to secondary tumor development thereby affecting clinical outcome of patients [14]. In untreated patients with cervical cancer, CTC subpopulations were classified based on EMT markers and a population with mesenchymal features was more common in patients with pelvic lymph node metastasis [15]. However, data on CTCs in patients with vulvar cancer are rare and a systematical characterization of CTCs during therapy which may be responsible for relapse in patients with vulvar or cervical cancers is missing.

We previously reported that various therapeutic approaches differentially affected the immune cell composition in the blood of patients with vulvar and cervical cancer. We showed that T-helper(Th)-17 and Tc17 cells remained in the blood of patients with vulvar or cervical cancer after radio- or chemoradiotherapy [16, 17]. In contrast, aRT in patients with vulvar cancer reduced the numbers of Th1 and CD8^+^ cytotoxic killer cells that additionally exhibited enhanced PD-1 expression after therapy. Consequently, this therapy-induced imbalance of immune cell composition was linked with vulvar cancer recurrence [17]. As CTCs represent a part of the systemic cellular milieu, we now prospectively evaluated numbers of cytokeratin^+^ CD45^−^ cells during stage-dependent therapy of patients with vulvar or cervical cancer via immunofluorescence and flow cytometry and analyzed their quantitative and phenotypical characteristics. In both tumor entities, we linked pre-therapeutic increased CTC numbers with advanced tumor FIGO stages, lymph node metastases and cancer relapse. We found varying CTC numbers in patients with vulvar cancer after aRT as well as in patients with cervical cancer after aCRT and pCRT and link the presence of post-therapeutic numbers of an identified PD-L1^+^ CD49f^+^ CD133^+^ CTC subpopulation within the therapy-induced systemic milieu with reduced recurrence free survival.

## Materials and methods

### Study participants and study design

Peripheral blood samples were obtained from 40 patients with vulvar cancer and 115 patients with cervical cancer before and during different therapeutic approaches as well as samples from untreated female age-matched immunocompetent controls (HC) and used for monitoring of CTC subsets constitution. A subpopulation of *n* = 40 age-matched HC were used in experiments for detection of CTCs via immunofluorescence (IF), respectively. In flow cytometry analysis a total of *n* = 115 HC were analyzed. Forty patients diagnosed with vulvar cancer were treated from 2017 to 2020 in the Saarland University Hospital. Within the 40 patients with vulvar cancer, 22 patients were treated with surgery alone, and 18 patients received surgery followed by adjuvant radiotherapy, of whom three received concurrent chemotherapy (cisplatin 40 mg/m^2^ body surface area). Results of these three patients are illustrated by black edging within the aRT cohort. Blood samples were obtained at primary diagnosis, one day after primary surgery, before the onset of aRT, and directly after completion of aRT. The applied irradiation in RT ranged from 45 to 66 Gy (Gy) (single dose 1.7–2.25 Gy). Forty patients were evaluated on follow-up.

One hundred fifteen patients diagnosed with cervical cancer were treated from 2017 to 2022 in the Saarland University Hospital. Within the 115 patients with cervical cancer, 57 patients were treated with surgery alone, and 58 patients received chemoradiotherapy (*n* = 36 adjuvant chemoradiotherapy (aCRT) and *n* = 22 primary chemoradiotherapy (pCRT)). Blood samples were obtained at primary diagnosis, one day after primary surgery, before the onset of platinum-based CRT, and directly after completion of CRT. The applied irradiation in CRT consisted of external beam irradiation (EBRT) (*n* = 44) or combined with intracavitary/interstitial HDR-brachytherapy (*n* = 14) and ranged from 45 to 59.92 Gy (Gy) for EBRT and 10–28 Gy for local brachytherapy. Concurrent chemotherapy was platinum-based, consisted of cisplatin (40 mg/m² body surface area) or carboplatin (AUC2) and was applied once a week with a maximum total number of six cycles. In follow-up studies, 50 patients out of 58 CCRT-treated patients could be included. Clinicopathologic and demographic data for the patient and HC cohorts are listed in Tables 1 and 2.

**Table 1:** Clinicopathologic characteristics of patients. Clinicopathologic characteristics of healthy controls and patients with vulvar cancer. aRT: adjuvant radiotherapy

Patients with vulvar cancer			
**Participants (n)**	**Healthy controls (n = 40)**	**Treated with surgery (n = 22)**	**Treated with aRT (n = 18)**
Age of diagnosis/participation (mean ± SD)	61.0 ± 5.7	63.9 ± 15.2	62.8 ± 11.7
Diagnosis:	/		
Squamous cell carcinomas (n = 40)		*n* = 22 (55%)	*n* = 18 (45%)
Tumor stage	/		
T1: *n* = 34 (84%)		*n* = 20 (50%)	*n* = 14 (35%)
T2: *n* = 5 (13%)		*n* = 2 (5%)	*n* = 3 (7%)
T3: *n* = 1 (3%)		/	*n* = 1 (3%)
Nodal stage	/		
N0: *n* = 27 (68%)		*n* = 22 (55%)	*n* = 5 (13%)
N1: *n* = 7 (17%)		*n* = 0 (0%)	*n* = 7 (17%)
N2: *n* = 6 (15%)		*n* = 0 (0%)	*n* = 6 (15%)
Stage (FIGO)	/		
I: *n* = 23 (58%)		*n* = 18 (45%)	*n* = 5 (13%)
II: *n* = 7 (17%)		*n* = 4 (10%)	*n* = 3 (7%)
III: *n* = 9 (22%)		*n* = 0 (0%)	*n* = 9 (22%)
IV: *n* = 1 (3%)		*n* = 0 (0%)	*n* = 1 (3%)
Grading	/		
G1: *n* = 3 (8%)		*n* = 3 (7%)	*n* = 0 (0%)
G2: *n* = 20 (50%)		*n* = 13 (32%)	*n* = 7 (17%)
G3: 15 (37%)		*n* = 5 (13%)	*n* = 10 (25%)
Data unavailable: *n* = 2 (5%)		*n* = 1 (3%)	*n* = 1 (3%)
Radiotherapy	/	**/**	
mean dose in Gray			51.1
range			45–66 Gy
concurrent chemotherapy			*n* = 3 (cisplatin 40 mg/m² body surface area)

**Table 2:** Clinicopathologic characteristics of patients. Clinicopathologic characteristics of healthy controls and patients with cervical cancer. CCRT: concurrent chemoradiotherapy; aCRT: adjuvant chemoradiotherapy; pCRT: primary chemoradiotherapy. HDR: high dose-rate

Patients with cervical cancer						
**Participants (n)**	**Healthy controls** **(n = 115)**	**Healthy controls subgroup for IF (n = 40/115)**	**Patients subgroup for IF (n = 40/115)**	**Treated with surgery (n = 57)**	**Treated with CCRT** **(n = 58)**	
					**aCRT (n = 36)**	**pCRT (n = 22)**
Age of diagnosis/participation (mean ± SD)	53.0 ± 8.6	52.0 ± 8.9	50.3 ± 11.0	50.2 ± 11.8	51.0 ± 13.4	55.1 ± 8.6
Diagnosis:	/	/				
Squamous cell carcinomas (n = 88)			*n* = 29 (33%)	*n* = 44 (50%)	*n* = 24 (27%)	*n* = 20 (23%)
Adenocarcinomas (*n* = 27)			*n* = 11 (41%)	*n* = 13 (48%)	*n* = 12 (44%)	*n* = 2 (8%)
Stage (FIGO)	/	/				
Stage I: *n* = 47 (40%)				*n* = 39 (33%)	*n* = 8 (7%)	*n* = 0 (0%)
I			*n* = 1	*n* = 1		
IA			*n* = 2	*n* = 2		
IA1			*n* = 5	*n* = 11		
IA2			*n* = 1	*n* = 3		
IB			*n* = 1	*n* = 4		
IB1			*n* = 7	*n* = 16	*n* = 6	
IB2			*n* = 2	*n* = 2	*n* = 2	
Stage II: *n* = 42 (37%)				*n* = 18 (16%)	*n* = 18 (16%)	*n* = 6 (5%)
II			*n* = 1	*n* = 3		
IIA			*n* = 1	*n* = 11		
IIA1			*n* = 3		*n* = 2	
IIA2			*n* = 3	*n* = 4	*n* = 3	*n* = 1
IIB					*n* = 13	*n* = 5
Stage III: *n* = 16 (14%)			*n* = 3		*n* = 10 (9%)	*n* = 6 (5%)
III					*n* = 3	*n* = 1
IIIA			*n* = 3			*n* = 2
IIIB					*n* = 6	*n* = 1
IIIC					*n* = 1	*n* = 2
Stage IV: *n* = 10 (9%)						*n* = 10 (9%)
IV			*n* = 3			*n* = 4
IVA			*n* = 1			*n* = 3
IVB			*n* = 3			*n* = 3
Nodal stage	/	/				
N0: *n* = 68 (59%)			*n* = 23	*n* = 56 (48%)	*n* = 1 (1%)	*n* = 11 (10%)
N1: *n* = 46 (40%)			*n* = 17	*n* = 0 (0%)	*n* = 35 (30%)	*n* = 11 (10%)
NX: *n* = 1 (1%)			*n* = 0	*n* = 1 (1%)	*n* = 0 (0%)	*n* = 0 (0%)
Chemoradiotherapy						
External beam irradiation					*n* = 36 (31%)	*n* = 8 (7%)
mean dose in Gray					51.7	53.6
range					50.4–59	45-59.9
intracavitary HDR brachytherapy						*n* = 14 (12%)
mean dose in Gray						21.6
range						10–28

### Detection of CTCs via Immunofluorescence (IF)

22 ml heparinized peripheral blood samples were collected and separated by Pancoll (Pan Biotec) density gradient centrifugation [18] to remove erythrocytes and granulocytes. PBMC numbers/ml blood was determined by trypan blue staining. 100,000 cells from PBMCs layer were seeded on glass microscope slides with reaction wells (Marienfeld, Lauda-Königshofen, Germany). After 30 min, cells were washed, fixed with 2% (wt/vol) paraformaldehyde in phosphate-buffered saline (PBS) for 15 min and permeabilized in 0.2% (vol/vol) Triton X-100 for 1 min. Cells were rinsed three times with PBS and incubated in 1% (wt/vol) bovine serum albumin (BSA) in PBS for 45 min. Cells were stained for leukocytes marker CD45 using rabbit anti-human CD45 polyclonal antibody (AB_442810, Abcam) and monoclonal mouse anti-human cytokeratin (AB_2892089, DAKO) followed by Alexa Fluor^®^ 488- (AB_2534088) or Alexa Fluor^®^ 546-conjugated secondary antibody (AB_2534093) (both Invitrogen) and 4′,6-diamidino-2-phenylindole (DAPI) staining. For visualization, a ZEISS Axio Imager KMAT with ZEN3.1 software from Zeiss was used. To evaluate CTC numbers, twelve independent pictures were taken per slide and numbers of DAPI^+^ cells as well as cytokeratin^+^ CD45^−^ cells were enumerated. Absolute numbers of CTCs/ml were determined using blood counts. Absolute numbers of CTCs/ml were calculated by incorporating proportions of cytokeratin^+^ CD45^−^ cells per PBMC numbers/ml and proportions of PBMCs per absolute number of leukocytes/ml.

### Detection of CTCs via flow cytometry

Cells from PBMCs layer after Pancoll (Pan Biotec) density gradient centrifugation were fixed using BD Bioscience Cytofix/Cytoperm Kit and stained with anti-CD45-PE (AB_395875) to exclude leukocytes and anti-cytokeratin-AF647 (AB_2738318) for CTC detection. For CTC characterization, antibodies against CD133 (BB700, AB_2744202), SOX2 (AF488, AB_10894382), CD49f (BV421, AB_2872200) and PD-L1 (BV510, AB_2739943) (all from BD Bioscience) were used. Cells were analyzed by flow cytometry (FACSCantoII; BD Biosciences).

### Statistics

All statistical analyses were performed using the GRAPHPAD Prism 8 (GRAPHPAD Software) program. To evaluate the statistical differences between the analyzed groups, a Mann-Whitney U-test was applied for the comparison of nonparametric data between two groups and the Kruskal-Wallis test for comparison of nonparametric data of > 2 groups. Paired analyses of CTC levels were performed using the Wilcoxon matched pairs test in the surgery only cohort or pCRT cohort. A paired Friedman test was used to compare differences in CTC levels in the aRT or aCRT cohort before surgery, after surgery and after aRT or aCRT. Correlation between tumor International Federation of Gynecology and Obstetrics (FIGO) stages and the number of CTCs was done using Spearman rank correlation. Best cutoffs to discriminate patients regarding CTC frequencies and with or without recurrent cancers were identified by receiver operator characteristics (ROC) analysis and Youden’s index calculation. Comparison of survival curves between different groups were evaluated by a Kaplan-Meier survival curve with log-rank (Mantel-Cox) test. A p value < 0.05 was considered statistically significant.

## Results

**Numbers of cytokeratin** ^**+**^ **CD45** ^**−**^ **cells in the blood of patients with vulvar or cervical cancer increased during carcinogenesis and were associated with lymph node metastases and cancer recurrence.**

**Fig. 1:**
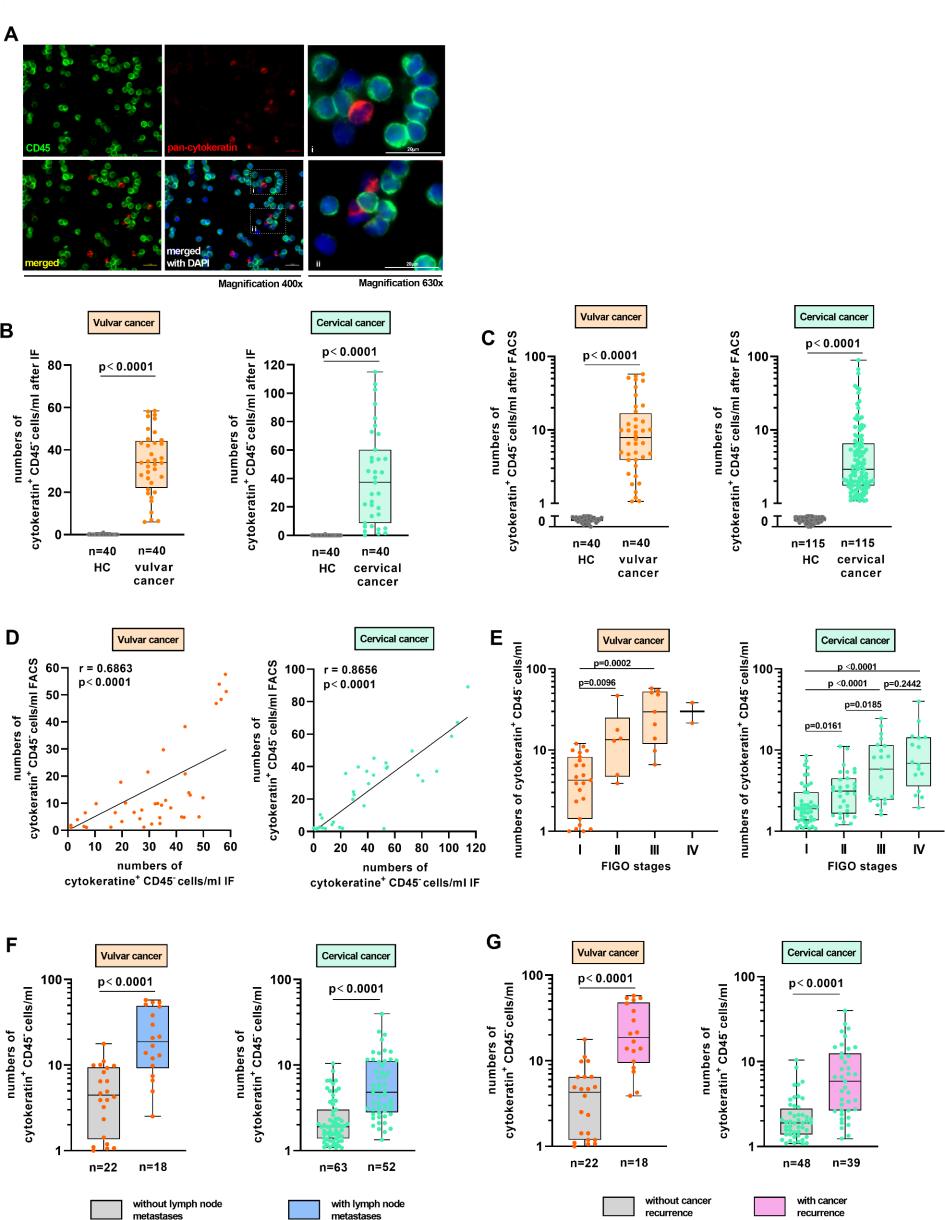
Cytokeratin^+^ CD45^−^ CTCs in the blood of patients with vulvar or cervical cancer before therapy. (**A**, **B**) Cells from PBMCs layers of 40 patients with vulvar cancer, 40 patients with cervical cancer and 40 female age-matched healthy controls (HC), respectively were analyzed by IF for CD45 (green) and pan-cytokeratin (red) expression. (**A**) shows representative pictures of a probe from a patients with cervical cancer. (**B**) Numbers of cytokeratin^+^ CD45^−^ cells/ml were evaluated for patients with vulvar cancer (orange dots), patients with cervical cancer (green dots) and HC (grey dots), respectively. (**C**) Cells from PBMCs were evaluated by flow cytometry for CD45 and pan-cytokeratin expression. Numbers of cytokeratin^+^ CD45^−^ cells/ml were evaluated for 40 patients with vulvar cancer (orange dots), 115 patients with cervical cancer (green dots) and 40 or 115 HC (grey dots), respectively. (**D**) Correlation of cytokeratin^+^ CD45^−^ cell numbers/ml after IF and flow cytometry of patients with vulvar cancer (orange dots) and patients with cervical cancer (green dots). (**E**) Correlation of cytokeratin^+^ CD45^−^ cell numbers/ml after flow cytometry of patients with vulvar cancer (orange dots) and patients with cervical cancer (green dots) with tumor FIGO stages. (**F**, **G**) Numbers of cytokeratin^+^ CD45^−^ cells/ml after flow cytometry in 40 patients with vulvar cancer (orange dots) and 115 or 87 patients with cervical cancer, respectively (green dots) (**F**) with (blue background) and without (grey background) lymph node metastases, (**G**) with (purple background) and without (grey background) cancer recurrence. P value according to the nonparametric Mann-Whitney U-test (**B**, **C**, **F**, **G**), nonparametric Kruskal-Wallis test (**E**) or Spearman rank correlation with linear regression (**D**)

We analyzed peripheral blood specimens obtained from 40 patients with vulvar cancer, a subgroup of 40 out of 115 cervical cancer patients (Tables 1 and 2), and 40 age-matched female HC, respectively via immunofluorescence (IF) stainings in initial experiments for CTC detection. In IF stainings of cells from PBMC layers, we found pan-cytokeratin positive cells (Fig. 1A; red) lacking the leukocyte marker CD45 (green). Evaluation of numbers of pan-cytokeratin^+^ CD45^−^ cells (CTCs) revealed a range of 6–58 CTCs/ml in untreated patients with vulvar cancer (Fig. 1B; orange dots) and a range of 1-115 CTCs/ml in untreated patients with cervical cancer (Fig. 1B; green dots). After detection of CTCs via IF, flow cytometry was used for further characterization. Using flow cytometry, CTCs were identified as pan-cytokeratin^+^ CD45^−^ cells (Supplementary Figure S1). Evaluation of CTCs resulted in a range of 1–58 CTCs/ml in 40 untreated patients with vulvar cancer (Fig. 1C; orange dots) and a range of 1–89 CTCs/ml in 115 untreated patients with cervical cancer (Fig. 1C; green dots). CTC numbers in HC were < 1/ml by both detection methods (grey dots). Results of both detection methods significantly correlated with each other in *n* = 40 patients with vulvar cancer (Fig. 1D, orange dots; *r* = 0.6863; *p*<0.0001) and *n* = 40 patients with cervical cancer (green dots; *r* = 0.8656; *p*<0.0001).

We were interested in whether pre-therapeutic numbers of CTCs of patients with vulvar or cervical cancer were associated with the subsequent course of disease. Numbers of CTCs/ml evaluated by flow cytometry increased in advanced FIGO stages in patients with vulvar (Fig. 1E, *n* = 40, orange dots) or cervical cancer (*n* = 115, green dots). Significantly higher numbers of CTCs were found in the blood of patients with vulvar (median: 19/ml) or cervical cancer (median: 5/ml) with lymph node metastases (Fig. 1F, blue background) in comparison to patients without metastases (grey background; median of 4/ml or 2/ml, respectively). Furthermore, patients who developed relapse on follow-up exhibited significantly higher numbers of CTCs (median of 19/ml for vulvar cancer or 6/ml for cervical cancer) (Fig. 1G, purple background) in comparison to patients without cancer recurrence (grey background; median of 4/ml or 2/ml, respectively). Thus, we detected pan-cytokeratin^+^ CD45^−^ CTCs in the blood of patients with vulvar and cervical cancer whose presence was linked with course of disease.

### Characterization of numbers of cytokeratin+ CD45− cells in the blood of patients with vulvar or cervical cancer during radio- and chemoradiotherapy

**Fig. 2:**
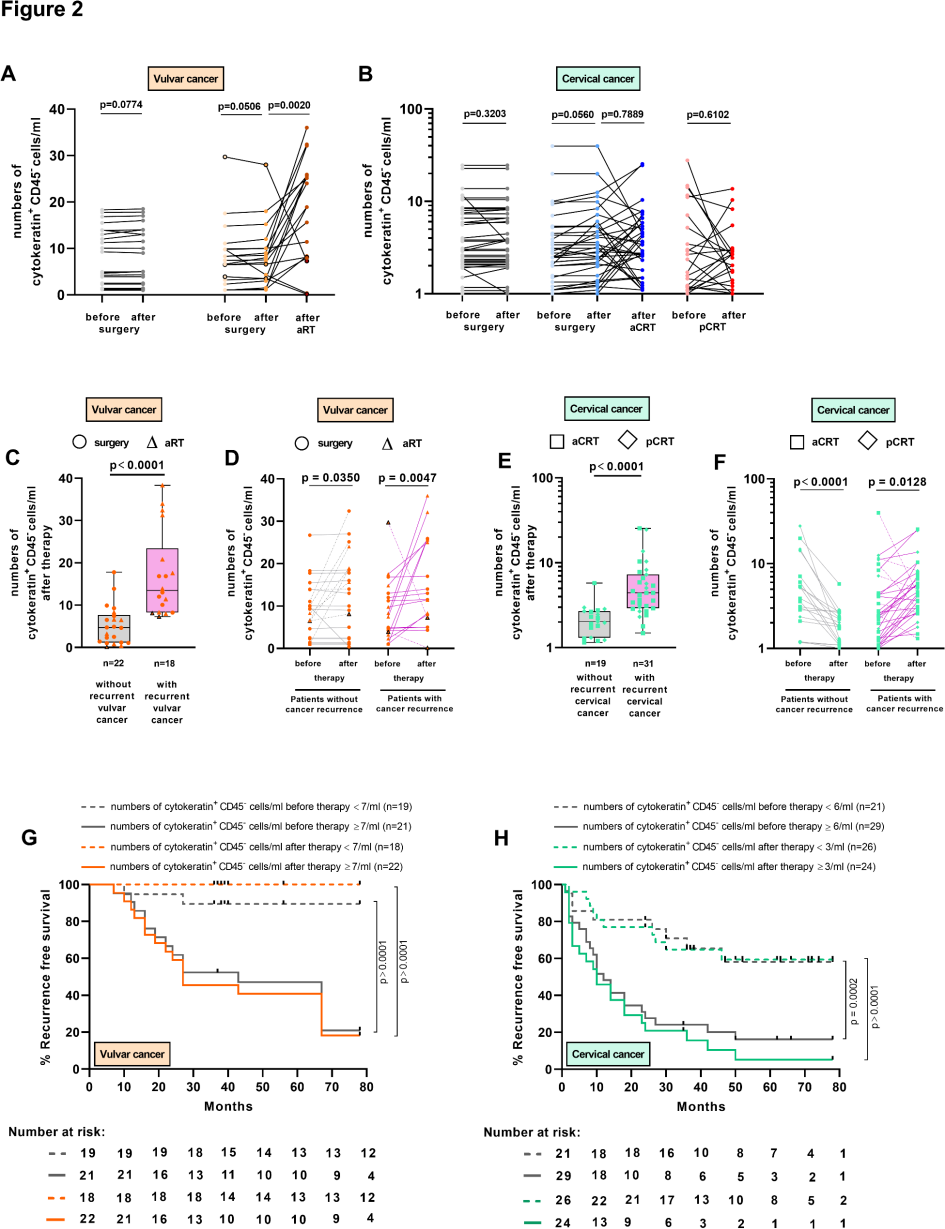
Impact of surgery, radiotherapy and chemoradiotherapy on CTCs in patients with vulvar or cervical cancer. (**A**) Cells from PBMCs of 40 patients with vulvar cancer (*n* = 22 receiving surgery, grey dots or *n* = 18 receiving aRT, orange dots; *n* = 3 patients with concurrent chemotherapy; black edged dots) and (**B**) cells from PBMCs of 115 patients with cervical cancer (*n* = 57 receiving surgery, grey dots, *n* = 36 receiving aCRT, blue dots or *n* = 22 receiving pCRT, red dots) were analyzed for CD45 and pan-cytokeratin expression by flow cytometry. (**C**, **D**, **E**, **F**) Numbers of post-therapeutic cytokeratin^+^ CD45^−^ cells/ml in patients with vulvar (orange dots) or cervical cancer (green dots) (**C**, **E**) with (purple background) and without (grey background) cancer recurrence, (**D**) before and after surgery (circles) or aRT (triangles) and (**F**) before and after aCRT (squares) and pCRT (diamond) with (purple lines) and without cancer recurrence (grey lines). P value according to the nonparametric matched pairs Wilcoxon test or paired Friedeman test (**A**, **B**, **D**, **F**) or Mann-Whitney U-test (**C**, **E**). (**G**) Recurrence-free survival (RFS) of 40 patients with vulvar cancer with pre-therapeutic numbers of cytokeratin^+^ CD45^−^ cells < 7/ml (dotted grey line) or ≥ 7/ml (grey line) as well as with post-therapeutic numbers < 7/ml (dotted orange line) or ≥ 7/ml (orange line). Median RFS was 35 months for pre-therapeutic numbers < 7/ml or 27 months for post-therapeutic numbers ≥ 7/ml. Comparison of survival analysis was performed using log-rank (Mantel-Cox) test; chi-square: 17.18 or 22.65, respectively. *P* < 0.0001. (**H**) RFS of 50 patients with cervical cancer with pre-therapeutic numbers of cytokeratin^+^ CD45^−^ cells < 6/ml (dotted grey line) or ≥ 6/ml (grey line) as well as with post-therapeutic numbers < 3/ml (dotted green line) or ≥ 3/ml (green line). Median RFS was 12 months for pre-therapeutic numbers ≥ 6/ml or 10 months for post-therapeutic numbers ≥ 3/ml. Comparison of survival analysis was performed using log-rank (Mantel-Cox) test; chi-square: 13.00 or 28.60, respectively, *P* = 0.0002 or *P* < 0.0001

Next, we were interested in whether therapeutic approaches modify numbers of CTCs. We evaluated numbers of pan-cytokeratin^+^ CD45^−^ cells in *n* = 40 patients with vulvar cancer treated with surgery alone (*n* = 22) or adjuvant RT (aRT) in addition to surgery (*n* = 18) as well as in *n* = 115 patients with cervical cancer treated with surgery alone (*n* = 57), surgery with aCRT (*n* = 36) and pCRT (*n* = 22). In patients with vulvar cancer, surgery only did not alter numbers of CTCs (Fig. 2A; grey dots), while after aRT numbers of CTCs significantly increased in total (orange dots). 15/18 patients showed an increase, and 3/18 patients a decrease in CTC numbers compared to pre-treatment levels.

In patients with cervical cancer (Fig. 2B), surgery did not significantly change numbers of CTCs in the surgery only (grey dots) as well as aCRT cohort (light and middle blue dots). However, after aCRT, half of patients (18/36) had increased CTC numbers and half of patients (18/36) exhibited decreased CTC numbers compared to pre-treatment baseline levels (middle and dark blue blots). Furthermore, 10/22 patients showed increased CTC numbers after pCRT while in 12/22 patients their numbers decreased compared to pre-treatment stages (light and dark red blots).

### Increased post-therapeutic cytokeratin+ CD45− cell numbers were associated with cancer relapse

As we found heterogeneous CTC numbers in patients with vulvar or cervical cancer after aRT, aCRT and pCRT, we evaluated the association of post-therapeutic CTC numbers with relapse. Patients with vulvar cancer who prospectively developed cancer recurrence exhibited significantly higher numbers of CTCs after therapy (Fig. 2C; median: 13/ml; circles: surgery only, triangles: surgery and aRT; purple background) in comparison to patients without relapse (median: 5/ml; grey background). Monitoring of CTCs in patients with vulvar cancer during therapy revealed that 7/22 patients without relapse exhibited an increase in CTC numbers after therapy (Fig. 2D, grey dotted lines) while 15/22 patients showed stable or decreasing CTC numbers (grey lines) compared to baseline. 16/18 patients with vulvar cancer who prospectively developed relapse showed increased post-therapeutic numbers, in only 2/18 patients CTC numbers were diminished (purple dotted lines).

Higher numbers of CTCs were also found in patients with cervical cancer who developed relapse (Fig. 2E; median: 5/ml; purple background) irrespective of treatment with aCRT (squares) or pCRT (diamonds). Interestingly, all 19 patients with cervical cancer without relapse showed a decrease in CTC numbers after aCRT and pCRT (Fig. 2F; grey lines; total reduction of 50%) whereas 25/31 patients (81%) who developed cancer recurrence on follow-up (purple lines) exhibited an increase in CTC numbers after aCRT and pCRT (56% in total). Only 6/31 patients with relapse showed a decrease in CTC numbers (dotted lines) whereas CTC levels remained at ≥ 2 CTCs/ml in 5 of these 6 patients.

To evaluate whether the presence of CTCs before and after therapy is linked to the course of disease, receiver operator characteristics (ROC) analysis was performed to identify a cut-off value for numbers of cytokeratin^+^ CD45^−^ CTCs that discriminates between patients with and without recurrent cancers. Forty patients (*n* = 22 after surgery (dots) and *n* = 18 (triangles) after surgery and aRT) could be evaluated for vulvar cancer recurrence. The median follow-up over all was 67 months (range 7–78 months). In our prospective study, 18/40 patients developed vulvar cancer recurrence, including seven after surgery only and 11 after surgery and aRT. For patients with vulvar cancer, the best discrimination was obtained for a cut-off of pre-therapeutic numbers of CTCs ≥ 7/ml (88.89% sensitivity and 81.82% specificity; Supplementary Figure S2A) and post-therapeutic numbers of CTCs ≥ 7/ml (100% sensitivity and 72.73% specificity; Supplementary Figure S2B). The area under the ROC curve (AUC) value was 0.9028 and 0.9028, respectively. Applying the defined cut-off values for 40 patients with vulvar cancer, in the group of patients with pre-therapeutic numbers of CTCs < 7/ml (grey dotted line) 6.5-years recurrence free survival (RFS) rate was 89.5% and for patients with pre-therapeutic numbers of CTCs ≥ 7/ml (grey line) 21% (Fig. 2G). Regarding post-therapeutic CTC numbers, no patients with CTC numbers < 7/ml (orange dotted line) developed relapse, however, in the group of patients with numbers of CTCs ≥ 7/ml (orange line) 3-years and 6.5-years RFS rate was 45.5% or 18.2%, respectively.

Furthermore, 50 out of 58 patients with cervical cancer treated with CRT (*n* = 32 after aCRT (squares) and *n* = 18 (diamonds) after pCRT) could be evaluated for relapse. The median follow-up over all was 24 months (range 9–78 months). 31/50 patients developed relapse, 21 after aCRT and 10 after pCRT. For patients with cervical cancer, the best discrimination was obtained for a cut-off value of pre-therapeutic numbers of CTCs ≥ 6/ml (51.28% sensitivity and 95.83% specificity; Supplementary Figure S2C) and post-therapeutic numbers of CTCs ≥ 3/ml (74.19% sensitivity and 94.74% specificity; Supplementary Figure S2D). The area under the ROC curve (AUC) value was 0.8296 and 0.8837, respectively. Applying the defined cut-off values for 50 patients with cervical cancer, in the group of patients with pre-therapeutic numbers of CTCs < 6/ml (grey dotted line) and post-therapeutic numbers < 3/ml (green dotted line) 6.5-years RFS rate was 58.1% or 59.4% in patients, respectively (Fig. 2H). In contrast, patients with pre-therapeutic CTCs ≥ 7/ml (grey line) or post-therapeutic numbers of CTCs ≥ 3/ml (green line) 3-years RFS rate was 16.1% or 5.1%, respectively. In conclusion, we found varying CTC numbers during therapy in patients with vulvar or cervical cancer and identified an association between increased post-therapeutic CTC numbers and cancer recurrence.

### Increased frequencies of CD49f+ CD133+ cytokeratin+ CD45− cells after radio- and chemoradiotherapy in the blood of patients with vulvar or cervical cancer

**Fig. 3:**
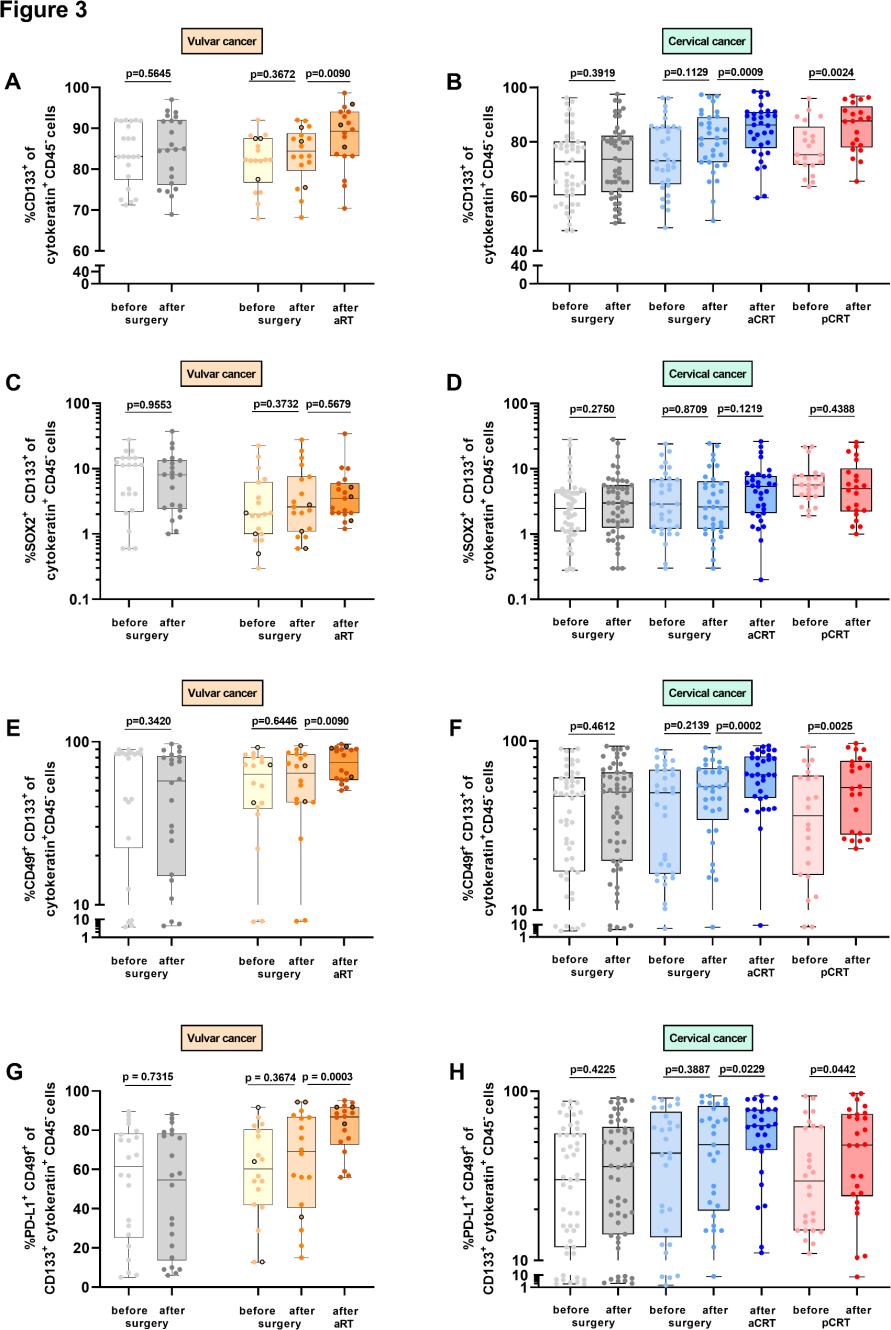
Phenotypic characterization of cytokeratin^+^ CD45^−^ cells during surgery, radiotherapy and chemoradiotherapy. Cells from PBMCs of 40 patients with vulvar cancer who received surgery only (*n* = 22; grey dots) or aRT (*n* = 18, orange dots; *n* = 3 patients with concurrent chemotherapy; black edged dots) and 115 patients with cervical cancer who received surgery only (*n* = 57; grey dots), aCRT (*n* = 36; blue dots) or pCRT (*n* = 22; red dots) were analyzed by flow cytometry for (**A**, **B**) frequencies of CD133^+^ cytokeratin^+^ CD45^−^ cells, (**C**, **D**) frequencies of SOX2^+^ CD133^+^ cytokeratin^+^ CD45^−^ cells, (**E**, **F**) frequencies of CD49f^+^ CD133^+^ cytokeratin^+^ CD45^−^ cells, (**G**, **H**) frequencies of PD-L1^+^ CD49f^+^ CD133^+^ cytokeratin^+^ CD45^−^ cells. P value according to the nonparametric Wilcoxon test or nonparametric Mann-Whitney U-test

We were interested in phenotypic characteristics of CTCs after therapy. The transmembrane glycoprotein CD133 is considered as stem cell marker and was found on CTCs of different tumor entities [19, 20]. In patients with vulvar or cervical cancer, 73-75% of CTCs were positive for CD133 before therapy (Fig. 3A, B; light grey dots). Surgery did not significantly alter percentages of CD133^+^ CTCs in the surgery only (grey dots), aRT (middle orange dots) or aCRT (middle blue dots) cohorts in patients with vulvar or cervical cancer (Fig. 3A, B). However, radiotherapy of patients with vulvar cancer (dark orange dots) as well as CRT of patients with cervical cancer (dark blue and red dots) significantly increased frequencies of CD133 resulting in 86-89% CD133^+^ CTCs.

Besides CD133, the stem cell marker SOX2 was found on CTCs [21]. In patients with vulvar or cervical cancer, 2-11.4% of CTCs were positive for SOX2 before therapy (Fig. 3C, D; light grey dots). In our experimental setup, surgery, radiotherapy as well as CRT did not significantly alter SOX2 expression on CD133^+^ CTCs in patients of both tumor entities (Fig. 3C, D).

Next, we evaluated the expression of CD49f (integrin α6), a known cervical cancer stem cell marker preferentially targeted by high risk HPVs [22] and found on CTCs of metastatic breast cancer patients [23]. In patients with vulvar or cervical cancer, 48.2-63.4% of CD133^+^ CTCs were positive for CD49f before therapy (Fig. 3E, F; light grey dots). Increased percentages of CD49f expressing CD133^+^ CTCs were found in patients with vulvar cancer after aRT (Fig. 3E; dark orange dots) as well as in patients with cervical cancer after aCRT (Fig. 3F; dark blue dots) and pCRT (dark red dots) in comparison to pre-therapeutic stages. Surgery only did not significantly alter CD49f expression on CTCs in our patients` cohorts (grey dots, light blue and middle blue dots). In conclusion, we identified increased frequencies of a subpopulation of CD49f^+^ CD133^+^ cytokeratin^+^ CD45^−^ CTCs in the blood of patients with vulvar or cervical cancer after adjuvant radiotherapy as well as adjuvant or primary chemoradiotherapy.

### Radio- and chemoradiotherapy increased PD-L1 expression on CTC subpopulations

As the PD-1/PD-L1 axis is a target of immunotherapy and antibodies against PD-1 are approved for the treatment of cervical cancers [24] as well as considered for the treatment of vulvar cancers [25, 26], we evaluated PD-L1 expression on CTCs during therapy. Pre-therapeutic frequencies ranged from 60.3 to 61.6% in our patients` cohorts, respectively. Percentages of PD-L1^+^ CD49f^+^ CD133^+^ CTCs increased after aRT in patients with vulvar cancer (Fig. 3G; dark orange dots; 1.3-fold) as well as after aCRT (Fig. 3H; dark blue dots; 1.3-fold) and pCRT (dark red dots; 1.6-fold) in patients with cervical cancer compared to baseline. Surgery did not alter PD-L1 expression of CTCs in the surgery only as well as aRT and aCRT cohorts (grey dots or light and middle blue dots) of both tumor entities. Thus, our results showed enhanced PD-L1 expression of CTCs after radio- or combined chemoradiotherapy.

### Post-therapeutic CTC subpopulations were associated with cancer relapse

**Fig. 4:**
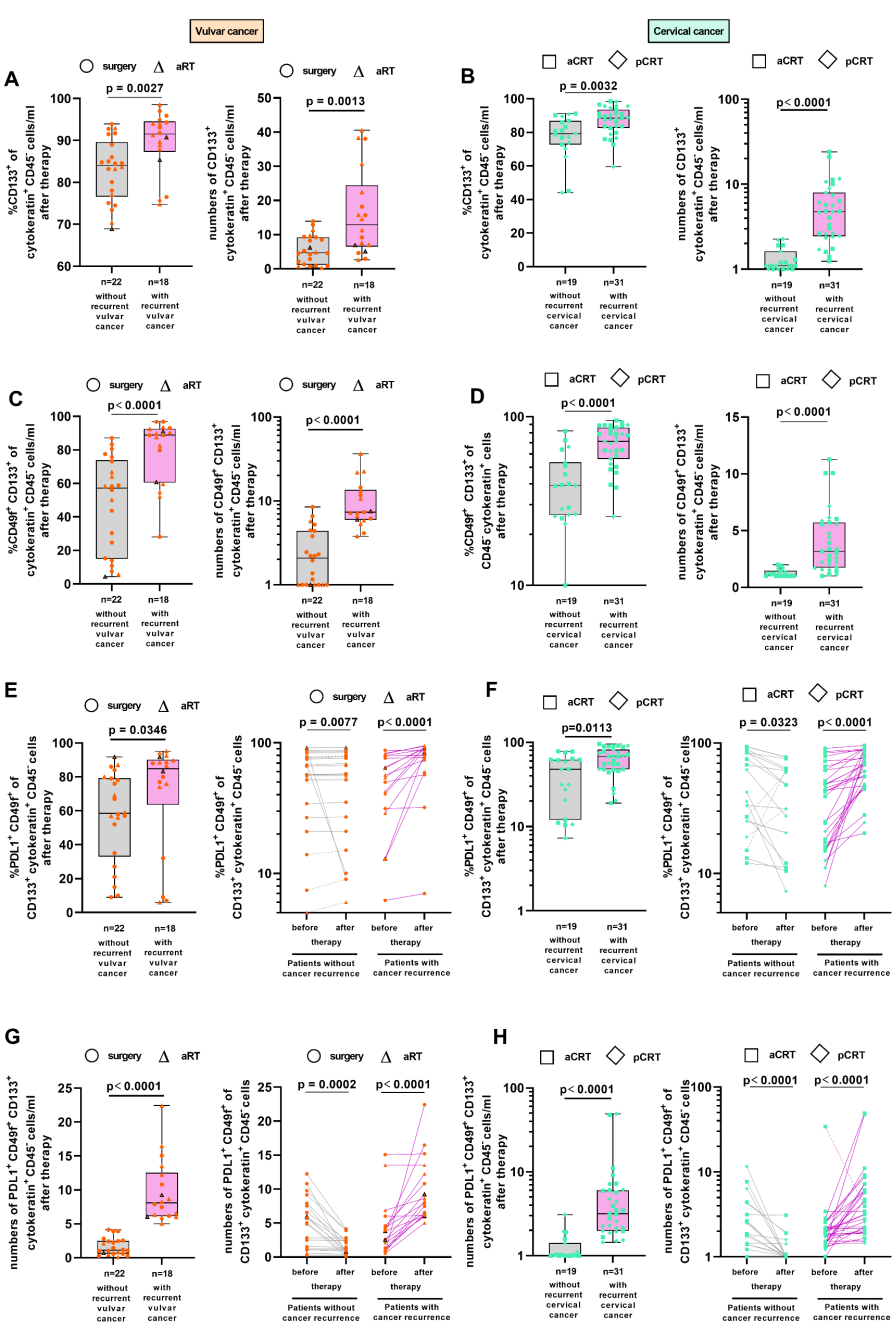
Frequencies and numbers of CTC subpopulations after therapy and their association with cancer relapse. Cells from PBMCs of 40 patients with vulvar cancer (orange dots) who received surgery only (*n* = 22; circles) or aRT (*n* = 18, triangles; *n* = 3 patients with concurrent chemotherapy; black edged triangles) and 50 patients with cervical cancer (green dots) who received aCRT (*n* = 36; squares) or pCRT (*n* = 22; diamonds) were analyzed for frequencies and numbers of (**A**, **B**) CD133^+^cytokeratin^+^ CD45^−^ cells, (**C**, **D**) frequencies and numbers of CD49f^+^ CD133^+^ cytokeratin^+^ CD45^−^ cells, (**E**, **F**, **G**, **H**) frequencies and numbers of PD-L1^+^ CD49f^+^ CD133^+^ cytokeratin^+^ CD45^−^ cells by flow cytometry. Frequencies and numbers were depicted for patients with (purple background) and without (grey background) relapse. P value according to the nonparametric Mann-Whitney U-test (**A**, **B**, **C**, **D**, **E** left, **F** left, **G** left, **H** left) or nonparametric Wilcoxon matched-pairs test (**E** right, **F** right, **G** right, **H** right)

To clarify the impact of CTC subpopulations after different therapeutic approaches in the blood of patients for clinical outcome, 40 patients after surgery and aRT could be evaluated for vulvar cancer recurrence and 50 patients after aCRT and pCRT for relapse of cervical cancer. Patients with recurrent vulvar or cervical cancer (purple boxes) exhibited higher percentages of CD133^+^ CTCs (Fig. 4A, B, left panels) and absolute numbers of CD133^+^ CTCs (right panels, respectively) in their blood after therapy than patients without relapse (grey boxes). The relevance of CD133 on CTCs for clinical outcome was underlined when we compared RFS in the absence or presence of CD133 on post-therapeutic CTC numbers (Supplementary S3A, B). Considering CD133 expression, all patients with vulvar cancer with numbers of CD133^+^ CTCs ≥ 14/ml developed relapse after 67 months, while numbers of pan-cytokeratin^+^ CD45^−^ CTCs ≥ 7/ml, without examination of CD133 expression, resulted in 6.5-year RFS rate of 18.2% as shown before (Fig. 2G; Supplementary S3A). In patients with cervical cancer, the presence of CD133 on CTCs numbers ≥ 2/ml after therapy was sufficient to mediate a better discrimination between patients with and without relapse resulting in differences in 6.5 years RFS rate of 73.7% (blue lines; Supplementary S3B). In comparison to pan-cytokeratin^+^ CD45^−^ CTCs, CD133^+^ CTC numbers ≥ 2/ml (blue line) led to comparable clinical outcome as post-therapeutic higher numbers of pan-cytokeratin^+^ CD45^−^ CTCs (> 3/ml, Supplementary S3B, green line).

Furthermore, significantly increased percentages (Fig. 4C, D, left panels) and absolute numbers of post-therapeutic CD49f^+^ CD133^+^ CTCs (Fig. 4C, D, right panels) were found in patients who developed vulvar or cervical cancer relapse. Solely CD49f expression on pan-cytokeratin^+^ CD45^−^ CTCs stratified patients with and without recurrent cancer (Supplementary S3D, E); however, a better discrimination was found when we considered concurrent CD49f and CD133 expression on CTCs (Fig. 4C, D) in comparison to solely CD49f expression on pan-cytokeratin^+^ CD45^−^ CTCs underlining the relevance of CD49f and CD133.

Analyzing the association of post-therapeutic PD-L1 expression on CTCs with cancer recurrence, we found that patients with vulvar (Fig. 4E, left panel) or cervical cancer (Fig. 4F, left panel) with relapse exhibited significantly higher percentages of PD-L1^+^ CD49f^+^ CD133^+^ CTCs in their blood (purple background) in comparison to patients without recurrence (grey boxes). Notably, all patients with vulvar (Fig. 4E, right panel; *n* = 18; purple lines) or cervical cancer (Fig. 4F; right panel; *n* = 31; purple lines) who developed relapse showed an increase in percentages of PD-L1^+^ CD49f^+^ CD133^+^ CTCs during therapy (purple lines; surgery (circle), aRT (triangle), aCRT (squares) and pCRT (diamonds)). In contrast, in patients without recurrence, only 4/18 patients with vulvar cancer (grey dotted lines) showed an increase in PD-L1^+^ CD49f^+^ CD133^+^ CTC frequencies while 14/18 patients consistently showed decreasing frequencies. For patients with cervical cancer without relapse, only 3/19 patients (grey dotted lines) showed increased frequencies compared to baseline while 16/19 patients exhibited a decrease in PD-L1^+^ CD49f^+^ CD133^+^ CTC frequencies (grey lines).

A better discrimination between patients with and without relapse was obtained when stratified for post-therapeutic PD-L1^+^ CD49f^+^ CD133^+^ CTC numbers/ml. Numbers of pan-cytokeratin^+^ CD45^−^ CTCs with solely PD-L1 expression after therapy were less efficient to discriminate patients with and without relapse (Supplementary S3F, G) than numbers of triple positive (PD-L1^+^ CD49f^+^ CD133^+^) CTCs (Fig. 4G, H). All patients with vulvar cancer with PD-L1^+^ CD49f^+^ CD133^+^ CTC numbers after therapy ≥ 5/ml developed cancer relapse (Fig. 4G; purple boxes). Interestingly, all 22 patients without vulvar cancer relapse exhibited a decrease in PD-L1^+^ CD49f^+^ CD133^+^ CTC numbers during therapy (Fig. 4G; right panel; grey lines) and all 18 patients with relapse showed an increase in numbers of this CTC subpopulation (purple lines).

Patients who developed relapse of cervical cancer in our prospective study (Fig. 4H; purple boxes) comprised significantly higher numbers/ml of this CTC subtype after therapy (median of 3/ml) than patients without recurrence (grey background; median of 1/ml). All patients without relapse showed decreasing PD-L1^+^ CD49f^+^ CD133^+^ CTC numbers during therapy (Fig. 4H, right, grey lines) while 28/31 patients (90%) with recurrent cancers exhibited increased post-therapeutic numbers/ml of this CTC subtype (purple lines) compared to baseline. In summary, we found a PD-L1^+^ CD49f^+^ CD133^+^ subpopulation of CTCs in patients with vulvar as well as cervical cancer whose presence was increased during therapy and was associated with cancer recurrence.

### Association between post-therapeutic CTCs with recurrence free survival

**Fig. 5:**
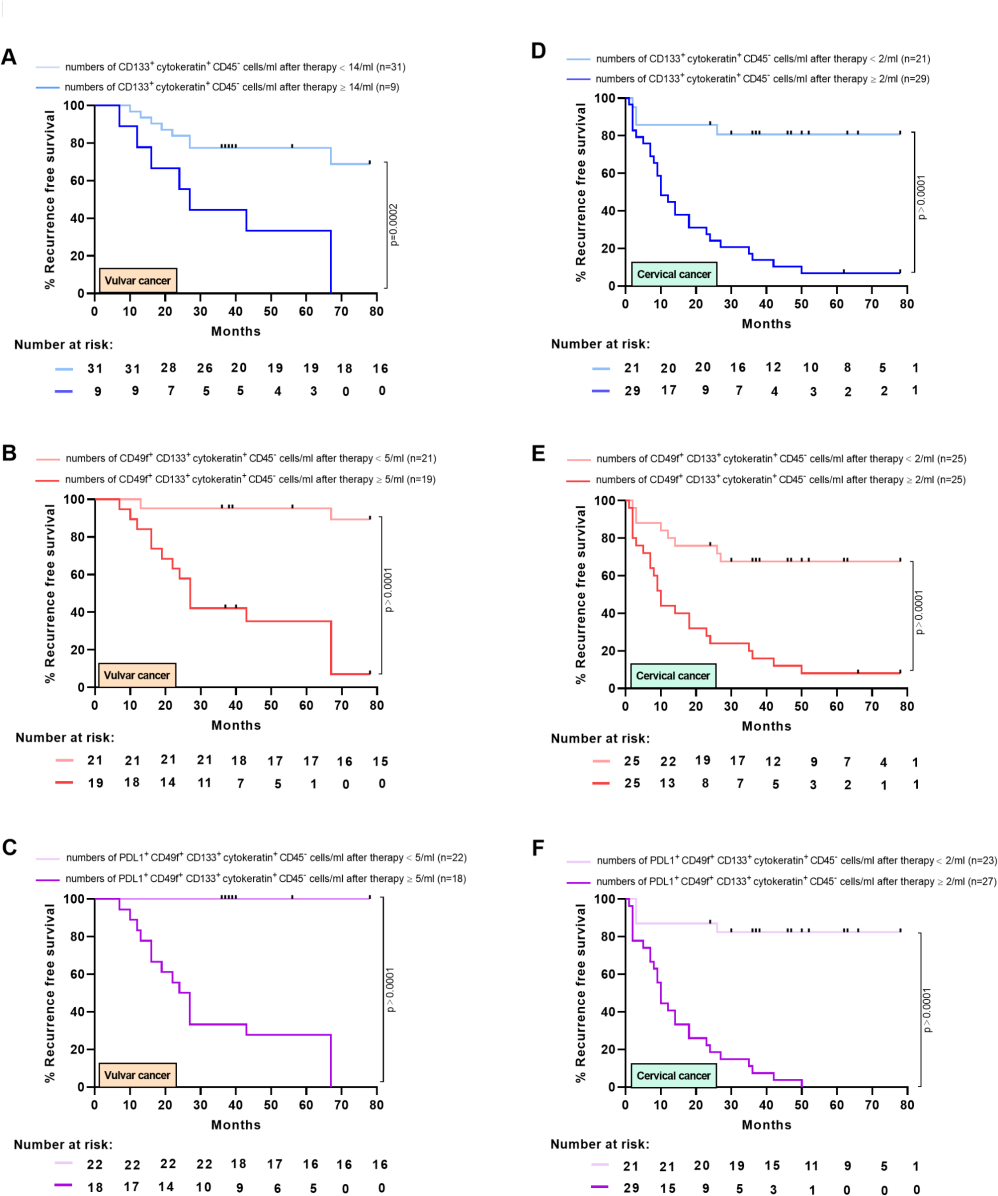
Association of numbers of CTC subpopulations with RFS of patients with vulvar or cervical cancer. (**A**) Recurrence-free survival (RFS) of 40 patients with vulvar cancer who received surgery (*n* = 22) or aRT (*n* = 18) was evaluated for a cohort with post-therapeutic numbers of CD133^+^ CTCs < 14/ml (light blue line) or ≥ 14/ml (blue line). Median RFS was 27 months for CTCs ≥ 14/ml. Comparison of survival analysis was performed using log-rank (Mantel-Cox) test; chi-square: 14.21, *P* = 0.0002. (**B**) RFS was evaluated for post-therapeutic numbers of CD49f^+^ CD133^+^ CTCs < 5/ml (light red line) or ≥ 5/ml (red line). Median RFS was 27 months for CTCs ≥ 5/ml. Comparison of survival analysis was performed using log-rank (Mantel-Cox) test; chi-square: 24.60, *P* < 0.0001. (**C**) RFS was evaluated for post-therapeutic numbers of PD-L1^+^ CD49f^+^ CD133^+^ CTCs < 5/ml (light purple line) or ≥ 5/ml (purple line). Median RFS was 25.5 months for CTCs ≥ 5/ml. Comparison of survival analysis was performed using log-rank (Mantel-Cox) test; chi-square: 41.26, *P* < 0.0001. (**D**) RFS for 50 patients with cervical cancer who received aCRT (*n* = 36) or pCRT (*n* = 22) was evaluated for post-therapeutic numbers of CD133^+^ CTCs < 2/ml (light blue line) or ≥ 2/ml (blue line). Median RFS was 10 months for CTCs ≥ 2/ml. Comparison of survival analysis was performed using log-rank (Mantel-Cox) test; chi-square: 23.60, *P* < 0.0001. (**E**) RFS was evaluated for post-therapeutic numbers of CD49f^+^ CD133^+^ CTCs < 2/ml (light red line) or ≥ 2/ml (red line). Median RFS was 10 months for CTCs ≥ 2/ml. Comparison of survival analysis was performed using log-rank (Mantel-Cox) test; chi-square: 17.83, *P* < 0.0001. (**F**) RFS was evaluated for post-therapeutic numbers of PD-L1^+^ CD49f^+^ CD133^+^ CTCs < 2/ml (light purple line) or ≥ 2/ml (purple line). Median RFS was 10 months for CTCs ≥ 2/ml. Comparison of survival analysis was performed using log-rank (Mantel-Cox) test; chi-square: 34.33, *P* < 0.0001

To evaluate whether the presence of CTC subtypes after therapy is linked to the course of disease, ROC analyses were performed to identify a cutoff value for numbers of the different subpopulations (CD133^+^ CTCs, CD49f^+^ CD133^+^ CTCs and PD-L1^+^ CD49f^+^ CD133^+^ CTCs) that discriminates between patients with and without recurrent cancers. For patients with vulvar cancer, the best discrimination was obtained for a cut-off value of post-therapeutic numbers of CD133^+^ CTCs ≥ 14/ml (50% sensitivity and 100% specificity; Supplementary Figure S4A), numbers of CD49f^+^ CD133^+^ CTCs ≥ 5/ml (88.89% sensitivity and 86.36% specificity; Supplementary Figure S44B) and numbers of PD-L1^+^ CD49f^+^ CD133^+^ CTCs ≥ 5/ml (100% sensitivity and 100% specificity; Supplementary Figure S4C). The AUC value was 0.7904, 0.9318 and 1.000, respectively. Applying the defined cut-off values for 40 patients with vulvar cancer, in the group of patients with numbers of CD133^+^ CTCs < 14/ml (light blue line, Fig. 5A), numbers of CD49f^+^ CD133^+^ CTCs < 5/ml (light red line, Fig. 5B) or numbers of PD-L1^+^ CD49f^+^ CD133^+^ CTCs < 5/ml (light purple line, Fig. 5C) after therapy, 6.5-year RFS rate was 68.8%, 90% or 100%, respectively. In contrast, patients with CD133^+^ CTCs ≥ 14/ml (blue line, Fig. 5A) or numbers of CD49f^+^ CD133^+^ CTCs ≥ 5/ml (red line, Fig. 5B), 3-years RFS rate was 44.4% or 42.1%, respectively. After 6.5-years, for patients with numbers of CD49f^+^ CD133^+^ CTCs ≥ 5/ml RFS was 7% (Fig. 5B), while all patients with CD133^+^ CTCs ≥ 14/m received relapse (blue line, Fig. 5A). For patients with numbers of PD-L1^+^ CD49f^+^ CD133^+^ CTCs ≥ 5/ml (purple line, Fig. 5C), 3-years RFS rate was 33.3% and after 67 months all patients exhibited recurrent vulvar cancer.

In patients with cervical cancer, the best discrimination was obtained for a cut-off value of post-therapeutic numbers of CD133^+^ CTCs ≥ 2/ml (83.87% sensitivity and 100% specificity; Supplementary Figure S3D), numbers of CD49f^+^ CD133^+^ CTCs ≥ 2/ml (76.67% sensitivity and 100% specificity; Supplementary Figure S3E) and numbers of PD-L1^+^ CD49f^+^ CD133^+^ CTCs ≥ 2/ml (80% sensitivity and 100% specificity; Supplementary Fig. S3F). AUC value was 0.9593, 0.9298 and 0.9763, respectively. Applying the defined cut-off values for 50 patients with cervical cancer after aCRT or pCRT, in the group of patients with numbers of CD133^+^ CTCs < 2/ml (light blue line, Fig. 5D), numbers of CD49f^+^ CD133^+^ CTCs < 2/ml (light red line, Fig. 5E) or numbers of PD-L1^+^ CD49f^+^ CD133^+^ CTCs < 2/ml (light purple line, Fig. 5F) after therapy, RFS after 78 months was 80.7%, 67.6% or 82.4%, respectively. In contrast, patients with CD133^+^ CTCs ≥ 2/ml (blue line, Fig. 5D) or numbers of CD49f^+^ CD133^+^ CTCs ≥ 2/ml (red line, Fig. 5E), 3-years RFS was 17.2% or 20%, respectively and 6.5-years RFS rate was 6.9% or 8%. For patients with numbers of PD-L1^+^ CD49f^+^ CD133^+^ CTCs ≥ 2/ml (purple line, Fig. 5F), 3-years RFS rate was 7.4% and after 50 months all patients exhibited recurrent cervical cancer. In conclusion, our data demonstrated a clear association between high numbers of PD-L1^+^ CD49f^+^ CD133^+^ CTCs in the blood of patients after therapy and vulvar or cervical cancer recurrence.

## Discussion

**Fig. 6:**
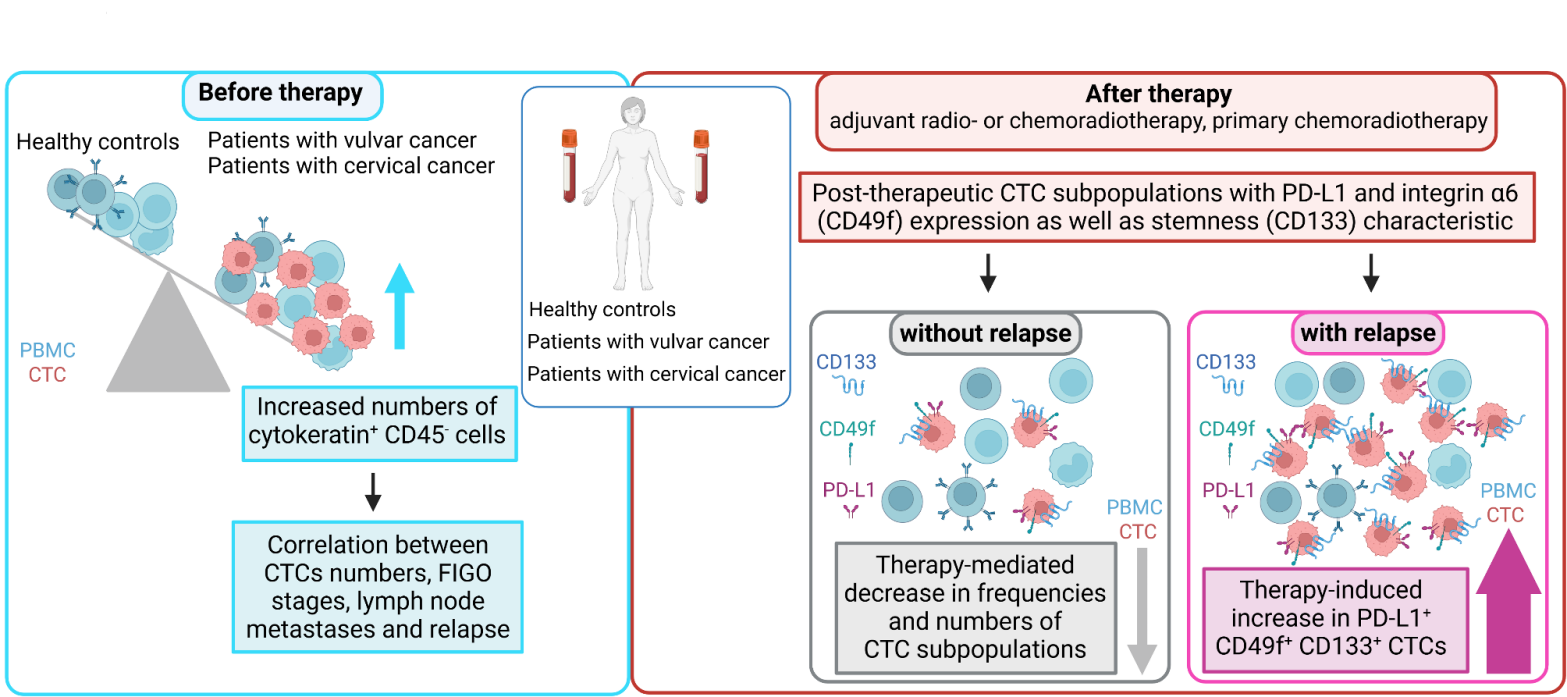
Schematic presentation of CTC subpopulations during therapy and vulvar cancer relapse. Adjuvant radio- or chemoradiotherapy increased occurrence of PD-L1^+^-CD49f^+^-CD133^+^ CTCs. Increased post-therapeutic numbers of PD-L1^+^-CD49f^+^-CD133^+^ CTCs were associated with vulvar or cervical cancer recurrence (scheme was generated with the BioRender software)

Disseminated cancer cells acquire the capability to become circulating tumor cells (CTCs) involved in cancer metastases. In this study, we investigated CTCs during vulvar cancer as well as cervical cancer stage-dependent therapy and showed that frequencies and absolute numbers of cytokeratin^+^ CD45^−^ CTCs individually differed in patients during therapy and linked therapy-induced increased CTC numbers with relapse on follow-up. Phenotypic characterization identified CTC subpopulations with stemness characteristics after therapy and a post-therapeutic PD-L1^+^ CD49f^+^ CD133^+^ CTC subpopulation in the patients’ blood of both tumor entities was associated with reduced recurrence free survival. Figure 6 summarizes our findings concerning CTC constitution during therapy of vulvar and cervical cancer patients.

The presence of CTCs in the patients’ blood before therapy is linked with poor prognosis in different tumor entities [13, 14, 27]. For cervical cancers, CTCs are described as cytokeratin^+^ CD45^−^ cells in the patients’ blood [13]. In line with previous results, pre-therapeutic CTC numbers of patients of our study increased during cervical carcinogenesis based on tumor FIGO stages [15] and were linked with lymph node metastases. For vulvar cancer patients, we also detected cytokeratin^+^ CD45^−^ cells and found an association with tumor FIGO stages and lymph node metastases. In patients of both tumor entities, pre-therapeutic CTC numbers were higher in patients who developed relapse on follow-up. Previous studies described different CTC subpopulations before therapy in patients with cervical cancers classified as epithelial CTCs, mesenchymal CTCs or mixed phenotype based on EMT markers [15]. Thereby, mesenchymal CTCs were more common in patients with pelvic lymph node metastasis and deep stromal infiltration [15]. However, post-therapeutic CTCs are involved in clinical course of disease and crucial to initiate metastases. Thus, monitoring is needed and knowledge on characteristics of CTCs during therapy of vulvar or cervical cancer patients is still missing.

Guideline-based therapy represents first line treatment for vulvar and cervical cancers. During therapy, adjuvant radio- or chemoradiotherapy are applied to target cancer cells [4, 8]. However, individual therapy responses cause individual course of diseases. The 5-year survival rates were indicated with only 25–41% in patients with metastatic vulvar [12] or less than 20% in metastatic cervical cancers [10]. Monitoring of CTCs during vulvar or cervical cancer stage-dependent therapies of this study revealed a differential impact of various therapeutic approaches on cytokeratin^+^ CD45^−^ CTCs resulting in individual decreases or increases in cytokeratin^+^ CD45^−^ CTC numbers during aRT, aCRT or pCRT compared to baseline levels, but not after surgery. Previous studies described increased numbers of viable circulating tumor cells in non-small cell lung cancer [28, 29], and bladder cancer [30] after radiotherapy as well as occurrence of CTCs with partial epithelial and mesenchymal phenotype in patients with breast cancer after chemotherapy [27]. In our study, patients who developed relapse on follow-up showed predominantly an increase in CTC numbers while in patients without relapse CTCs numbers declined. Notably, the presence of post-therapeutic cytokeratin^+^ CD45^−^ CTCs was linked with worse RFS in comparison to pre-therapeutic cytokeratin^+^ CD45^−^ CTCs underlining the relevance for consideration of systemic therapy-induced changes of CTCs.

To survive systemically in the patients’ blood after leaving primary tumors, CTCs must obtain the capability to resist against attacks from the immune system as well as therapy-induced killing. Here, evaluation of phenotypic characteristics of CTCs during therapy identified enhanced frequencies and numbers of CTC subpopulations exhibiting PD-L1 and integrin α6 (CD49f) expression as well as stemness characteristics. We found increased frequencies of pentaspan transmembrane glycoprotein CD133^+^ CTCs after radio- and chemoradiotherapy that was described on cancer stem-like cells of different cancer types [31]. CD133 favors signaling of tumor-promoting pathways, like PI3K and Wnt/β-catenin and was associated with long-term self-renewal of cells [31]. In line with previous results demonstrating that CD133^+^ CTCs predicted poor progression-free survival in patients with metastatic castration‐sensitive prostate cancer [19], our results stated worse recurrence-free survival for patients when we considered CD133^+^ cytokeratin^+^ CD45^−^ in comparison to cytokeratin^+^ CD45^−^ CTCs. It is assumed that CD133 is linked to further stem-cell marker expression, like SOX2 [31]. High SOX2 expression in cervical cancer tissues was associated with radiation resistance of patients [32]. In our study, expression of SOX2 by CTCs of patients with vulvar or cervical cancer was detectable; however, SOX2 expression on CTCs was not enhanced by radio- or chemoradiotherapy.

In addition to stem cell marker expression, integrin α6 (CD49f) was found on CTCs of patients from this study. Integrins, as cellular adhesion molecules, play key roles in cancer progression including survival of CTCs, priming of the metastatic niche, extravasation into the secondary site and metastatic colonization of the new tissue [33]. CD49f was found on CTCs of patients with metastatic breast cancer [23]. In cervical carcinogenesis, CD49f is assumed as a marker of cervical cancer stem cells potentially linked with CD133 and SOX2 expression [34] and preferentially targeted by high-risk HPVs [22]. CD49f expression was found at the invasive front of cervical cancer spheroids and cervical cancer tissues associated with cancer metastasis [35]. In this study, our data clearly showed increased frequencies and numbers of CD49f^+^ CD133^+^ CTCs after aRT as well as aCRT and pCRT compared to baseline predominantly in those patients who developed relapse on follow-up. As CD49f was associated with multi drug resistance and prognosis in ovarian cancer [36] and tamoxifen resistance in breast cancer cells [37], CD49f together with CD133 may help CTCs to systemically survive cancer therapies. This assumption could by substantiated by our data demonstrating that CD49f^+^ CD133^+^ double positive CTCs superiorly stratified patients with and without vulvar and cervical cancers than solely CD49f^+^ CTCs.

PD-1-PD-L1-interactions allow cancer cells to avoid immune cell mediated killing. In line with CTCs from non-small cell lung cancer [38], urothelial carcinoma [39] or prostate cancer [40], CTCs from patients with vulvar or cervical cancer exhibited pre-therapeutic PD-L1 expression. After aRT, aCRT or pCRT, frequencies and numbers of PD-L1^+^ CD49^+^ CD133^+^ CTCs increased predominantly in patients who developed relapse. Notably, best discrimination was obtained between patients with and without recurrent cancer when patients were stratified for post-therapeutic PD-L1^+^ CD49^+^ CD133^+^ CTC numbers ≥ 5 or ≥ 2/ml, respectively. This CTC subpopulation was directly linked with reduced recurrence-free survival and superior to solely PD-L1^+^ CTCs regarding discrimination between patients with and without relapse.

To contribute to cancer metastases, CTCs must survive within and thereby interact with the systemic therapy-induced individual milieu of patients. We previously described that aRT in patients with vulvar cancer as well as aCRT and pCRT in patients with cervical cancer affected the immune cell composition. We showed that Th17 cells and Tc17 cells systemically survived radio- or chemoradiotherapy [16, 17] while Th1 cells and perforin-producing CD8^+^ killer cells (CTLs) declined [17]. Interestingly, Th1 and CTLs exhibited increased PD-1 expression after therapy which was associated with relapse [17]. Thus, therapy-induced PD-1 expression by Th1 and CTLs may provide a target for PD-L1^+^ CTCs preventing CTL-mediated killing. Furthermore, IL-17 is described to promote spheroid formation and self-renewal of CD133^+^ cancer stem-like cells in ovarian cancer [41]. As frequencies of IL-17 expressing CD4^+^ and CD8^+^ T cells systemically increased after therapy in patients with vulvar or cervical cancer [16, 17], interaction of CTCs and IL-17 may favor CTC survival and their contribution to metastases. Our study is limited as longitudinal analysis concerning stability of therapy-induced CTC phenotypes is lacking. Furthermore, a direct comparison of CTC numbers evaluated by other studies remains difficult due to different methods used for CTC detection and low sample size of patients with vulvar cancer representing a potential weakness. Nevertheless, differences in therapy-induced CTC subtypes between patients with and without cancer relapse of both tumor entities are very pronounced and correlate well with the severity of the disease indicating the need for new treatment approaches, especially for patients who do not respond to standard treatments.

Notably, with our flow cytometry analysis we were able to directly detect protein expression of CTCs that might represent therapeutic targets. For vulvar cancer treatment, first studies indicated the relevance for immune checkpoint inhibitors, like the anti-PD-1 antibody pembrolizumab, especially in combination with radiotherapy [25, 26] and showed varying, but promising results with cancer remission and improved survival rates [42, 43]. For cervical cancers, pembrolizumab is approved as second line treatment in combination with a platinum-based chemotherapy in patients with PD-L1^+^ advanced, metastatic or recurrent cervical cancer targeting PD-L1-PD-1-axis [24]. Recent results from the KEYNOTE-826 trial stated that metastatic patients with high PD-L1 expression of cervical cancer tissues benefited from combined treatment with pembrolizumab and chemotherapy [44]. However, patients with reccurent or metastatic cervical cancers with PD-L1 expression scores less than 1% also profited by combined anti-PD-1 (cemiplimab) and chemotherapy treatment [45]. Thus, based on our results, clinical monitoring of post-therapeutic PD-L1 profile of CTCs should be taken into account besides PD-L1 expression scores on cancer tissues as potential basis for second line treatment decision to assist monitoring of early relapse and improve prospects of PD-L1-PD-1 immunotherapy. Furthermore, blockage of CD49f was tested as therapeutic approach identifying pranlukast, a drug used to treat asthma, as inhibitor of CD49f preventing adhesion of breast cancer cells to laminin, mammosphere formation and CD49f-dependent PI3K activation [46]. In addition, anti-human integrin α6-blocking antibody P5G10 induces apoptosis in primary acute lymphoblastic B-cell leukemia (ALL) cells and sensitizes these cells for chemotherapy in an ALL mouse model [47, 48].

## Conclusion

Our study identified a PD-L1^+^ CD49^+^ CD133^+^ subpopulation of circulating tumor cells in patients with vulvar or cervical cancer and linked their post-therapeutic occurrence with relapse. Based on our studies, it should be clarified how the PD-L1^+^ CD49^+^ CD133^+^ CTC phenotype associated with poor clinical outcome develops during carcinogenesis within the tumormicromilieu, survives cancer therapies and can be systemically sustained potentially supported by the therapy-induced systemic cellular or soluble milieu. Our results further suggest evaluation of CD113, CD49f and PD-L1 as target for (immuno)therapy in vulvar and cervical cancers.

## Electronic supplementary material

Below is the link to the electronic supplementary material.

Supplementary Material 1

## Data Availability

The original contributions presented in the study are included in the article/supplementary materials. The data that support the findings of this study are available from the corresponding author upon reasonable request.

## Abbreviations

aCRT: Adjuvant chemoradiotherapy
aRT: Adjuvant radiotherapy
DAPI: 4′,6-diamidino-2-phenylindole
EMT: Epithelial-mesenchymal-transition
FIGO: International Federation of Gynecology and Obstetrics
HC: Healthy controls
HPV: Human papillomavirus
IL: Interleukin
PBMC: Peripheral blood mononuclear cells
PI3K: Phosphoinositide 3-kinase
PD-1: Programmed Cell Death Protein
