# Supplementary material for: PD-L1+ CD49f+ CD133+ Circulating tumor cells predict outcome of patients with vulvar or cervical cancer after radio- and chemoradiotherapy
Source: J Transl Med. 2025 Mar 13;23:321. doi: 10.1186/s12967-025-06277-w (PMC11908062; doi:10.1186/s12967-025-06277-w)
Supplement: Supplementary file 1 — Supplementary Material 1 [file 12967_2025_6277_MOESM1_ESM.pdf]

PD-L1+ CD49f+ CD133+ circulating tumor cells predict outcome of vulvar and cervical cancer patients after radio- and chemoradiotherapy.

Selina Gies, Patrick Melchior, Istvan Molnar, Gregor Olmes, Russalina Stroeder, Tanja Tänzer, Maike Pohlers, Moritz Schäfer, Laura Theobald, Martina Sester, Erich Franz Solomayer, Barbara Walch-Rückheim

Table of contents:

Supplementary Figure S1: Gating strategies for quantification of CTC populations.

Supplementary Figure S2: ROC analysis of CTCs before therapy.

Supplementary Figure S3: Association of numbers of CTC subpopulations with recurrence free survival of patients with vulva and cervical cancer.

Supplementary Figure S4: ROC analysis of CTC subpopulations after therapy.

Supplementary Figure S1

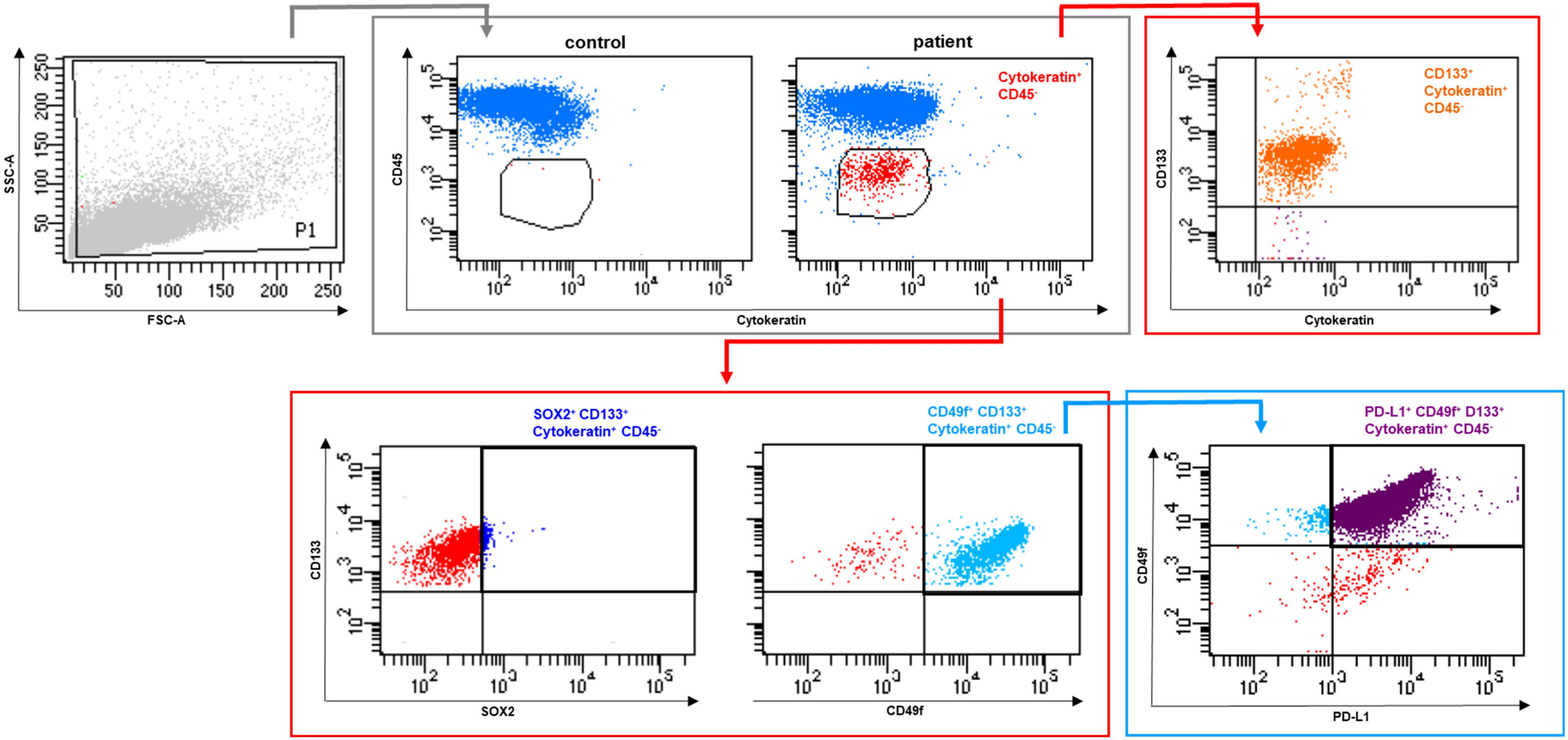

Supplementary Figure S1: Gating strategies for quantitation of CTC populations.
To quantify CTC populations, cells from PBMC layers were gated within all events according to SSC-A and FSC-A. Among all cells excluding small cell debris, CTCs were identified according to cytokeratin expression and lack of lymphocyte marker CD45 expression (Cytokeratin+, CD45-). Cytokeratin+ CD45- cells were further evaluated for CD133 expression (orange), SOX2 and CD133 co-expression (dark blue) as well as CD49f and CD133 co-expression (light blue). CD49f+ CD133+ Cytokeratin+ CD45- cells were further evaluated for PD-L1 expression (purple). Data were analyzed on FACS Canto-II using DIVA Software-6.1.3 (BD Bioscience). Within the DIVA software, different subpopulations were depicted in particular colors in dot plots and used for final figure preparation using GRAPHPAD Prism 8 software.

Supplementary Figure S2

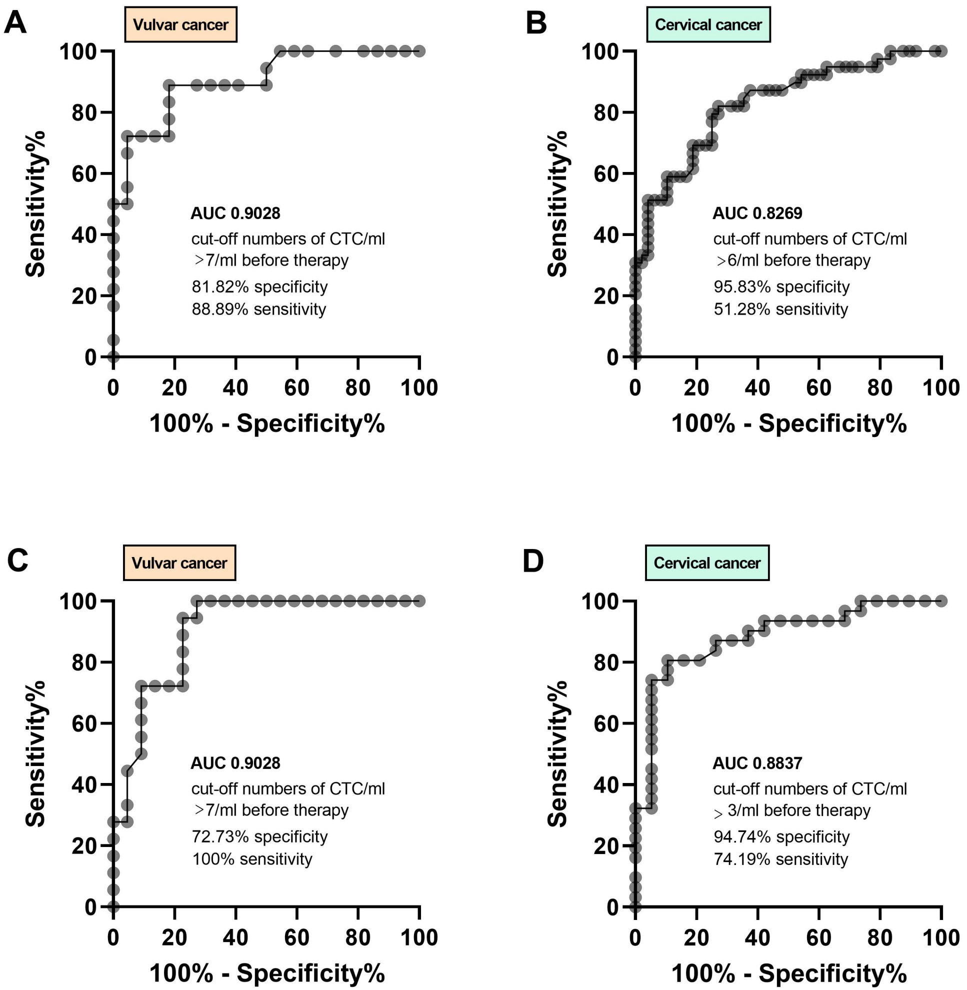

Supplementary Figure S2: ROC analysis of CTCs before therapy. ROC analysis of (A) CD45- cytokeratin+ CTCs before therapy in vulvar cancer patients, (B) ROC analysis of CD45- cytokeratin+ CTCs before therapy in cervical cancer patients, (C) ROC analysis of CD45- cytokeratin+ CTCs after therapy in vulvar cancer patients, (D) ROC analysis of CD45- cytokeratin+ CTCs after therapy in cervical cancer patients.

Supplementary Figure S3

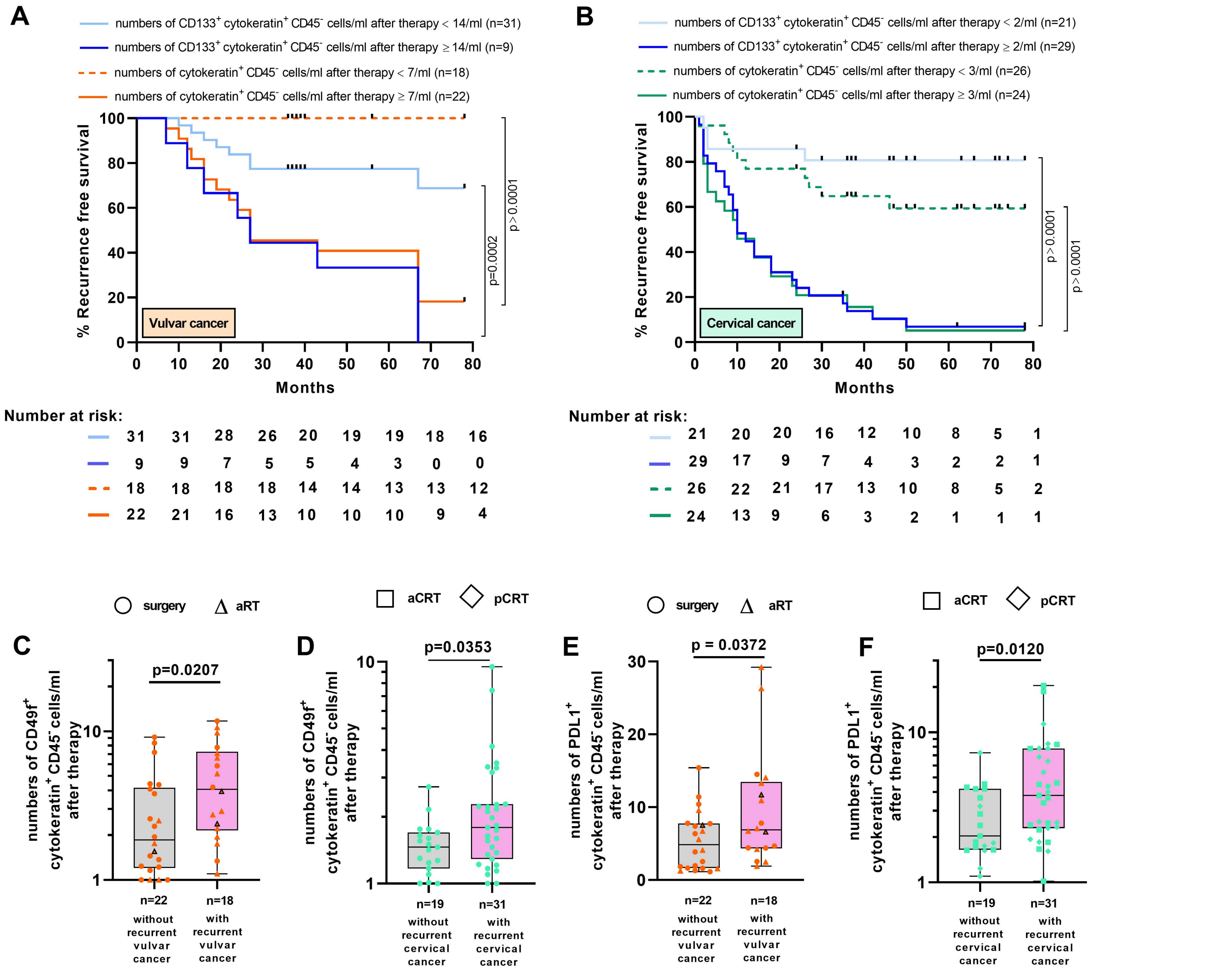

Supplementary Figure S3: Association of numbers of CTC subpopulations with recurrence free survival of patients with vulvar or cervical cancer. (A) Recurrence-free survival (RFS) of 40 patients with vulvar cancer who received surgery (n=22) or aRT (n=18) was evaluated for a cohort with post-therapeutic numbers of CD133$^+$ CTCs less than 14/ml (light blue line) or ≥ 14/ml (blue line). Median RFS was 27 months for the cohort with CTCs ≥ 14/ml. Comparison of survival analysis was performed using log-rank (Mantel-Cox) test; chi-square: 14.21, P=0.0002. Recurrence-free survival (RFS) of 40 patients with vulvar cancer was determined for a cohort with post-therapeutic numbers of cytokeratin$^+$ CD45$^-$ cells less than 7/ml (dotted orange line) or ≥ 7/ml (orange line). Median RFS was 35 months for the cohort with post-therapeutic numbers ≥ 7/ml. Comparison of survival analysis was performed using log-rank (Mantel-Cox) test; chi-square: 22.65. P < 0.0001. (B) RFS for 50 patients with cervical cancer who received aCRT (n=36) or pCRT (n=22) was evaluated for a cohort with post-therapeutic numbers of CD133$^+$ CTCs less than 2/ml (light blue line) or ≥ 2/ml (blue line). Median RFS was 10 months for the cohort with CTCs ≥ 2/ml. Comparison of survival analysis was performed using log-rank (Mantel-Cox) test; chi-square: 23.60, P < 0.0001. RFS of 50 patients with cervical cancer was determined for a cohort with post-therapeutic numbers of cytokeratin$^+$ CD45$^-$ cells less than 3/ml (dotted green line) or ≥ 3/ml (green line). Median RFS was 10 months for the cohort with post-therapeutic numbers ≥ 3/ml. Comparison of survival analysis was performed using log-rank (Mantel-Cox) test; chi-square: 28.60. P < 0.0001. (C, D, E, F) Cells from PBMCs of 40 patients with vulvar cancer (orange dots) who received surgery only (n=22; circles) or aRT (n=18, triangles; n=3 patients with concurrent chemotherapy; black edged triangles) and 50 patients with cervical cancer (green dots) who received aCRT (n=36; squares) or pCRT (n=22; diamonds) were analyzed for numbers of (C, D) CD49f$^+$ cytokeratin$^+$ CD45$^-$ cells and (E, F) PD-L1$^+$ cytokeratin$^+$ CD45$^-$ cells by flow cytometry. Frequencies and numbers were depicted for patients with (purple background) and without (grey background) relapse. P value according to the nonparametric Mann-Whitney U-test .

Supplementary Figure S4

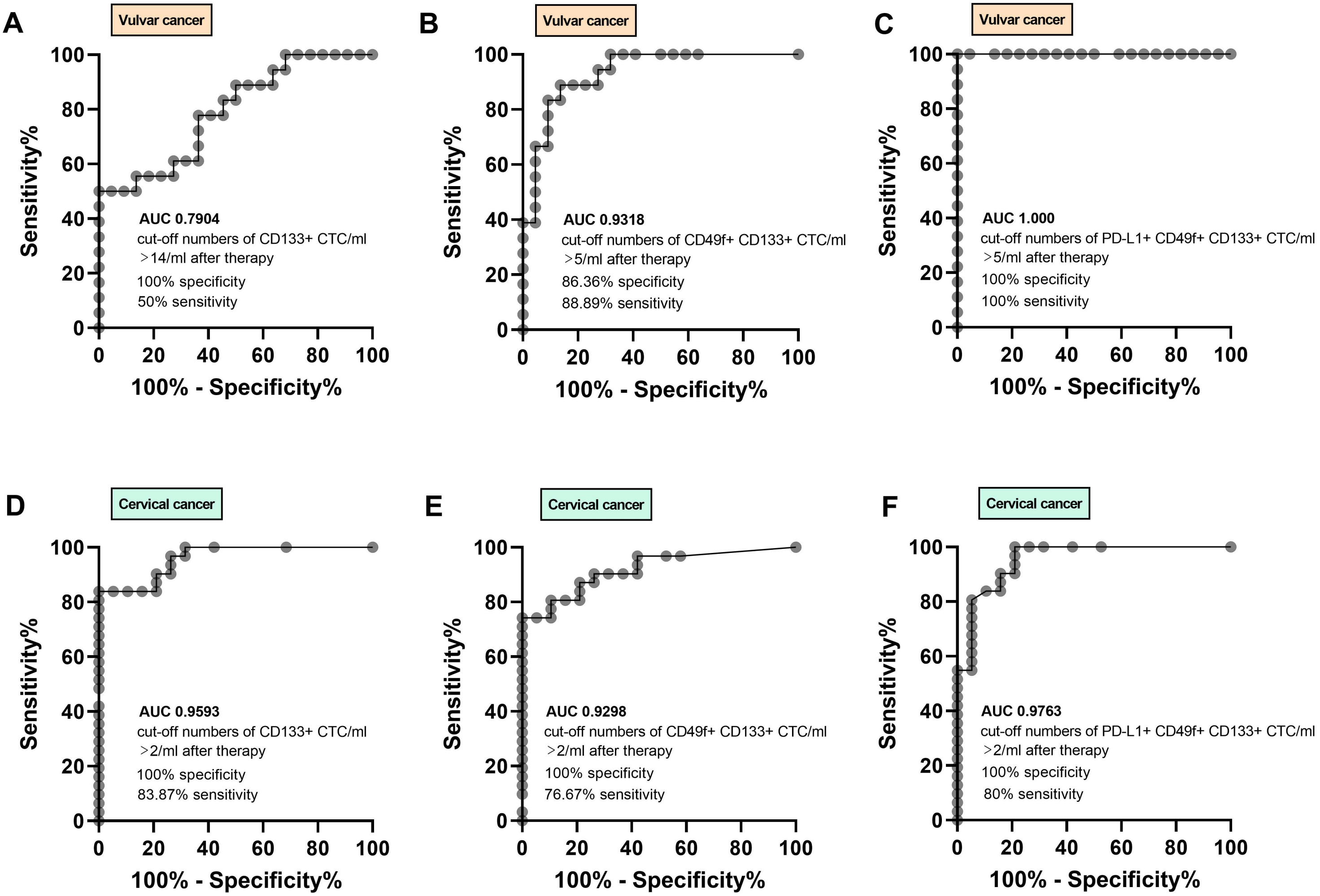

Supplementary Figure S4: ROC analysis of CTC subpopulations after therapy. ROC analysis of (A) CD133+ CTCs, (B) CD49f+ CD133+ CTCs and (C) PD-L1+ CD49f+ CD133+ CTCs after therapy in vulvar cancer patients. ROC analysis of (D) CD133+ CTCs, (E) CD49f+ CD133+ CTCs and (F) PD-L1+ CD49f+ CD133+ CTCs after therapy in cervical cancer patients.
